# Nitrogen fixation and transcriptome of a new diazotrophic *Geomonas* from paddy soils

**DOI:** 10.1128/mbio.02150-23

**Published:** 2023-10-19

**Authors:** Guo-Hong Liu, Shang Yang, Shuang Han, Cheng-Jie Xie, Xing Liu, Christopher Rensing, Shun-Gui Zhou

**Affiliations:** 1 Institute of Resources, Environment and Soil Fertilizer, Fujian Academy of Agricultural Sciences, Fuzhou City, Fujian Province, China; 2 Fujian Provincial Key Laboratory of Soil Environmental Health and Regulation, College of Resources and Environment, Fujian Agriculture and Forestry University, Fuzhou City, Fujian Province, China; Oregon State University, Covallis, Oregon, USA

**Keywords:** new diazotrophic *Geomonas*, nitrogen fixation, nitrogenase, transcriptome analysis, RT-qPCR, paddy soil

## Abstract

Nitrogen gas (N_2_) fixation driven by diazotrophs is a crucial process for supplying nitrogen to paddy soil ecosystems. The genus *Geomonas* has been considered to be an important potential diazotroph in paddy soils, but direct experimental evidence of the nitrogen-fixing ability of *Geomonas* in pure culture is still lacking. Hence, we aimed to demonstrate this nitrogen-fixing capability and shed light on how this process was regulated in response to ammonium (NH_4_^+^) in *Geomonas*. In this study, we determined that a key nitrogenase gene (*nifH*) was present in 50 isolates from paddy soils. Members of *Geomonas* contained the minimum nitrogen fixation gene cluster (*nifBHDKEN*) based on genomic analysis, implying *Geomonas* species had the potential to fix nitrogen. Acetylene reduction assay (ARA), ^15^N_2_ isotope labeling, and total nitrogen accumulation assays validated that *Geomonas* was, indeed, able to fix nitrogen in pure culture. Under nitrogen-fixing conditions, the cell morphology of *Geomonas* changed from short rod-shaped (with NH_4_^+^) to long rod-shaped and flagella became longer and thicker. The expression of genes correlated to nitrogen fixation in the *Geomonas* transcriptome was quantified in response to NH_4_^+^. Expression of genes associated with nitrogenase, flavin-based electron bifurcation complexes (such as the FixAB system), NH_4_^+^ uptake, and transformation (e.g., glutamine and glutamate synthetases) were significantly upregulated under nitrogen-fixing conditions, suggesting these mechanisms might be involved in N_2_ fixation in *Geomonas*. These results were verified by RT-qPCR. Taken together, our results demonstrate that *Geomonas* species possess the ability to fix N_2_ and expand our understanding on the ecological significance and potential applications of *Geomonas* in paddy soil ecosystems.

**IMPORTANCE:**

The ability of *Geomonas* species to fix nitrogen gas (N_2_) is an important metabolic feature for its application as a plant growth-promoting rhizobacterium. This research is of great importance as it provides the first comprehensive direct experimental evidence of nitrogen fixation by the genus *Geomonas* in pure culture. We isolated a number of *Geomonas* strains from paddy soils and determined that *nifH* was present in these strains. This study demonstrated that these *Geomonas* species harbored genes encoding nitrogenase, as do *Geobacter* and *Anaeromyxobacter* in the same class of *Deltaproteobacteria*. We demonstrated N_2_-dependent growth of *Geomonas* and determined regulation of gene expression associated with nitrogen fixation. The research establishes and advances our understanding of nitrogen fixation in *Geomonas*.

## INTRODUCTION

Biological nitrogen fixation (BNF) performed by diazotrophs is a critical process in ensuring sustainable food production for the growing global population while minimizing the environmental impact. BNF is one of the most important nitrogen cycling processes in rice ecosystems, converting atmospheric nitrogen gas (N_2_) into ammonium (NH_4_^+^) used by plants (1–3). Rice is the primary economic food crop for over half of the global population. To fulfill the food requirements for the growing global population, chemical nitrogen fertilizers had to be applied to rice crops to achieve high yields (4). Nevertheless, approximately 56% of the applied nitrogen fertilizer is lost to land and water or converted into nitrous oxide through denitrification (5), which leads to various environmental issues including soil acidification, heightened greenhouse gas emissions, and jeopardizing the sustainable development of agriculture and food security (6). BNF is friendly to the environment and nitrogen-fixing microorganisms mainly include symbiotic and free-living diazotrophs (7, 8). Compared to symbiotic diazotrophs, free-living diazotrophs possess the advantage of lacking host-specificity and are extensively distributed in various environments (9, 10). Recent studies have highlighted the significance of free-living diazotrophs in NH_4_^+^ production *in situ* and their crucial role in nitrogen fixation in rice fields (11, 12). Free-living diazotrophs are primarily composed of the phylum *Proteobacteria* (genus *Azotobacter*, *Klebsiella*, *Pseudomonas*, *Geobacter*, *Anaeromyxobacter*), as well as *Cyanobacteria*, *Firmicutes* (*Clostridium* and *Bacillus*), and *Actinobacteria* (2, 13, 14). These taxa have the ability to fix nitrogen and, thus, maintain the nitrogen requirements for plant growth when nitrogen sources become limited or absent. Currently, genome analysis has unveiled that a broad spectrum of bacteria harbor nitrogen fixation genes (*nif*), suggesting that the diversity of diazotrophs extends beyond what was previously recognized in terrestrial environments (15–17). Therefore, it is imperative to explore the potential significance of unknown diazotrophs and conduct further research to better comprehend their functional roles in supporting ecosystem health and productivity.

The genus *Geomonas* established by Xu et al. in 2019 displays a close genetic relationship with *Geobacter*, a member of the family *Geobacteraceae* of the class *Deltaproteobacteria. Geomonas* species have been shown to be strictly anaerobic and capable of iron reduction (16–21). Until now, there have been 16 validly published species of *Geomonas*, primarily found in paddy soils (22). Subsequent research has inferred that *Geomonas* may function as a novel type of potential diazotroph similar to *Geobacter* in rice soil ecosystems (16, 17, 19). Previous studies indicated that the presence of *nif* gene transcripts from *Deltaproteobacteria,* particularly from the genus *Geobacter* and *Anaeromyxobacter,* was frequently detected and had a high relative abundance in paddy soils (12, 23–26). Although the reports in this area have been limited, nitrogen-fixing activity has been demonstrated by culture-dependent methods in *Geobacter* (*Geobacter metallireducens* and *Geobacter sulfurreducens*) and *Anaeromyxobacter* spp. (2, 13, 27–30). As such, it is deduced that *Geomonas* may represent the dominant diazotrophs in the rice soil ecosystem. Consequently, it is crucial to explore the distribution of *Geomonas* in paddy soils and further study its nitrogen fixation properties at the culture-dependent level.

The primary objective of this study was to determine the nitrogen-fixing activity and gene regulatory changes in *Geomonas* under nitrogen-fixing conditions. We hypothesize that *Geomonas* is an important new diazotroph, playing a crucial role in fixing nitrogen in paddy soil. However, to date, there has been no comprehensive direct research on nitrogen fixation activity of *Geomonas* in pure culture. Therefore, demonstrating the diazotrophic activity in *Geomonas* is required for a comprehensive understanding of the microbial drivers of nitrogen cycling in soil environments. In this study, we conducted genomic, transcriptomic, and culture-dependent analyses on *Geomonas* strains isolated from paddy soils to verify their diazotrophy. This research contributes to a better understanding of the genetic mechanisms of diazotrophy in *Geomonas* as well as providing a scientific foundation for exploring anaerobic microbial ecology in rice fields.

## RESULTS

### Identification of diazotrophic *Geomonas* from paddy soils

Fifty *Geomonas* strains were isolated from paddy soils by cultural methods and identified using 16S rRNA gene sequences. Seven of them have been identified as five novel species of genus *Geomonas* through polyphasic taxonomy and validly published (16, 19). Based on 16S rRNA gene sequences analysis, other isolates were identified as six known species of the genus *Geomonas*, including *Geomonas terrae*, *Geomonas ferrireducens*, *Geomonas paludis*, *Geomonas oryzae*, *Geomonas edaphica*, and *Geomonas bremensis* (Fig. S1).

PCR results showed that all isolates displayed a *nifH* band with a fragment length of about 360 bp (Fig. S2), indicating that *Geomonas* potentially possessed the ability to fix nitrogen. To estimate the nitrogen-fixing ability, nitrogenase activity was measured in representative isolates by the acetylene reduction assay (ARA) method and showed that *Geomonas* displayed nitrogenase activity (Fig. S3). Among these isolates, a novel species, *Geomonas nitrogeniifigens* RF4, exhibited the highest nitrogenase activity and was used for further studies.

### Genomics and enzymatic background of nitrogenase in *Geomonas*

**Fig 1:**
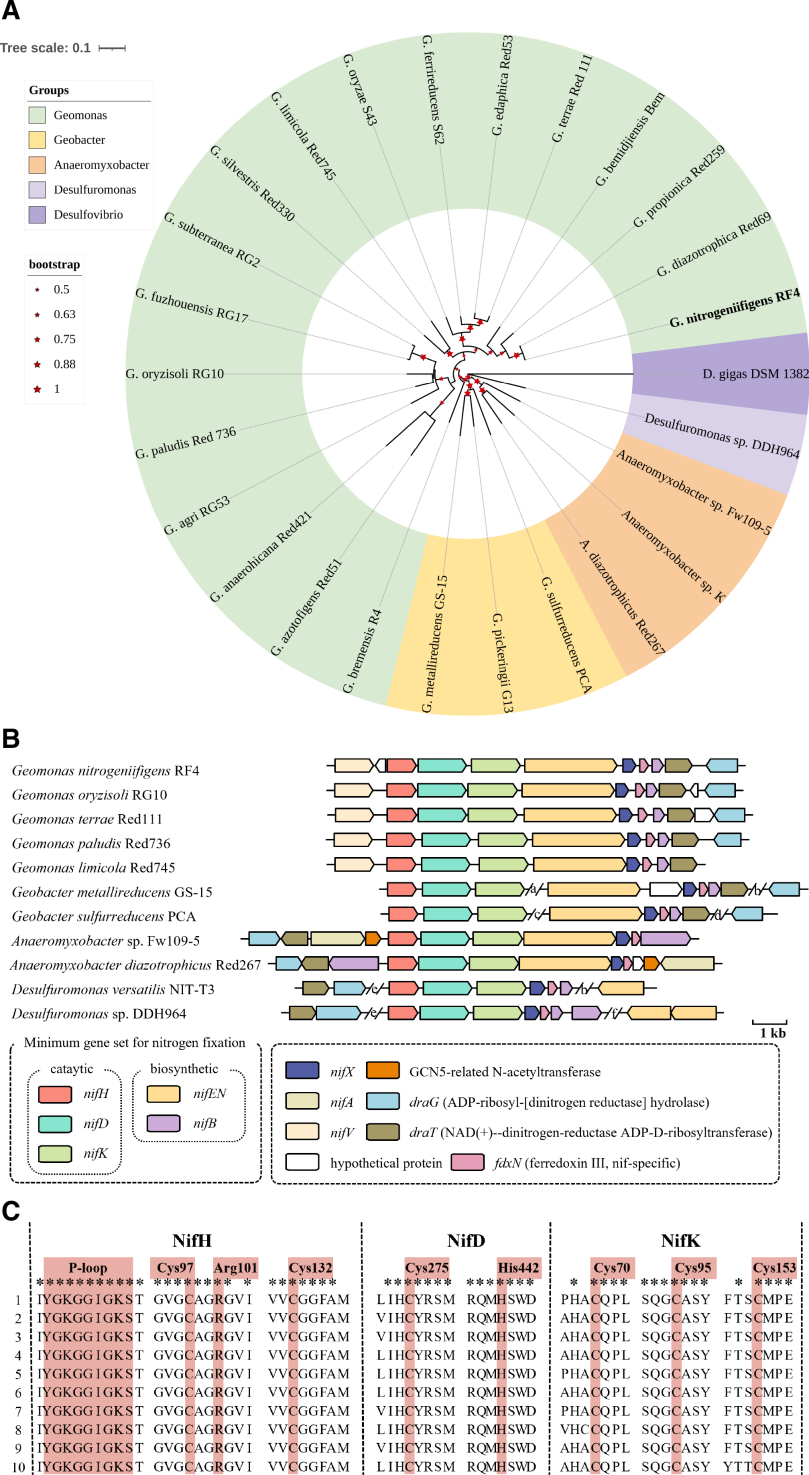
Phylogeny and nitrogenase gene regions in *Geomonas* based on genome analysis. (**A**) Neighbor-joining phylogenetic tree of the aligned amino acid sequences of NifHDK retrieved from genomes. (**B**) Comparison of the *nif* gene cluster in *Geomonas* and its relatives. Note: regions a to f contain 4, 5, 12, 1, 2, 8 genes, respectively. Homologous genes are shown in the same colors. (**C**) Alignments around the crucial residues of NifHDK in *Geomonas* and related diazotrophs. 1, *Geomonas nitrogeniifigens* RF4; 2, *Geomonas oryzisoli* RG10^T^; 3, *Geomonas terrae* Red 111^T^; 4, *Geomonas paludis* Red 736^T^; 5, *Geomonas limicola* Red745^T^; 6, *Geobacter metallireducens* GS-15^T^; 7, *Geobacter sulfurreducens* PCA^T^; 8, *Anaeromyxobacter diazotrophicus* Red267^T^; 9, *Anaeromyxobacter* sp. Fw109-5; 10, *Desulfuromonas versatilis* NIT-T3^T^; 10, *Desulfuromonas* sp. DDH964.

The genome size of the genus *Geomonas* was ranged between 4.46–5.26 Mb, and the number of protein-coding genes was 3,889–4,455. The genomic DNA G+C content was 60.3–62.6% (Table S1). The *nifHDK* nucleotide sequences retrieved from genomes in *Geomonas* showed the closest phylogenetic relationship to those in *Geobacter* and *Anaeromyxobacter* within the class *Deltaproteobacteria* (Fig. 1A).

Based on genome predictions, KEGG results showed that the genes *nifHDKENX* encoding functions associated with nitrogenase were found on sequenced genomes of *Geomonas. nifHDK* encoded the proteins of the nitrogenase complex, and one copy of *nifDK* and one to two copies of *nifH* were predicted in *Geomonas* (Fig. S4). Five genes adjacent to *nifH* (*nifHDKENX*) were conserved in the investigated *Geomonas nif* gene clusters (Fig. 1B). The *nif* gene cluster of *Geomonas* contained genes encoding regulatory proteins (*draG*, *fdxN,* and *draT*), nitrogenase complex structural genes (*nifHDK*), and biosynthesis of the FeMo cofactor (*nifBENX*). The *nif* gene cluster structure of *Geomonas* and *Geobacter* was different and even exhibited differences within the genus. Strains *G. nitrogeniifigens* RF4 and *G. terrae* Red111^T^ had a similar *nif* gene cluster that was different from *G. paludis* Red736^T^. Moreover, the structure of the *nif* gene cluster of the genus *Geomonas* was more compact than that of other genera in class *Deltaproteobacteria*, namely, these genes were located closer to each other in *Geomonas*.

Residues in alignments of NifH sequences showed that [4Fe-4S] iron sulfur cluster-ligating cysteines (Cys97 and Cys132) and P-loop/MgATP binding motif were conserved in *Geomonas* as well as in other diazotrophs (Fig. 1C), suggesting that these proteins might function as the dinitrogenase reductase. Similarly, *nifDK* sequences were conserved in these *Geomonas* species. The crucial residues of the FeMo cofactor binding site in NifD (Cys275 and His442) and in the P cluster in NifK (Cys70, Cys95, and Cys153) were conserved, as in other diazotrophs such as *Anaeromyxobacter* and *Geobacter*.

### Nitrogen fixation and growth responses of *Geomonas*

**Fig 2:**
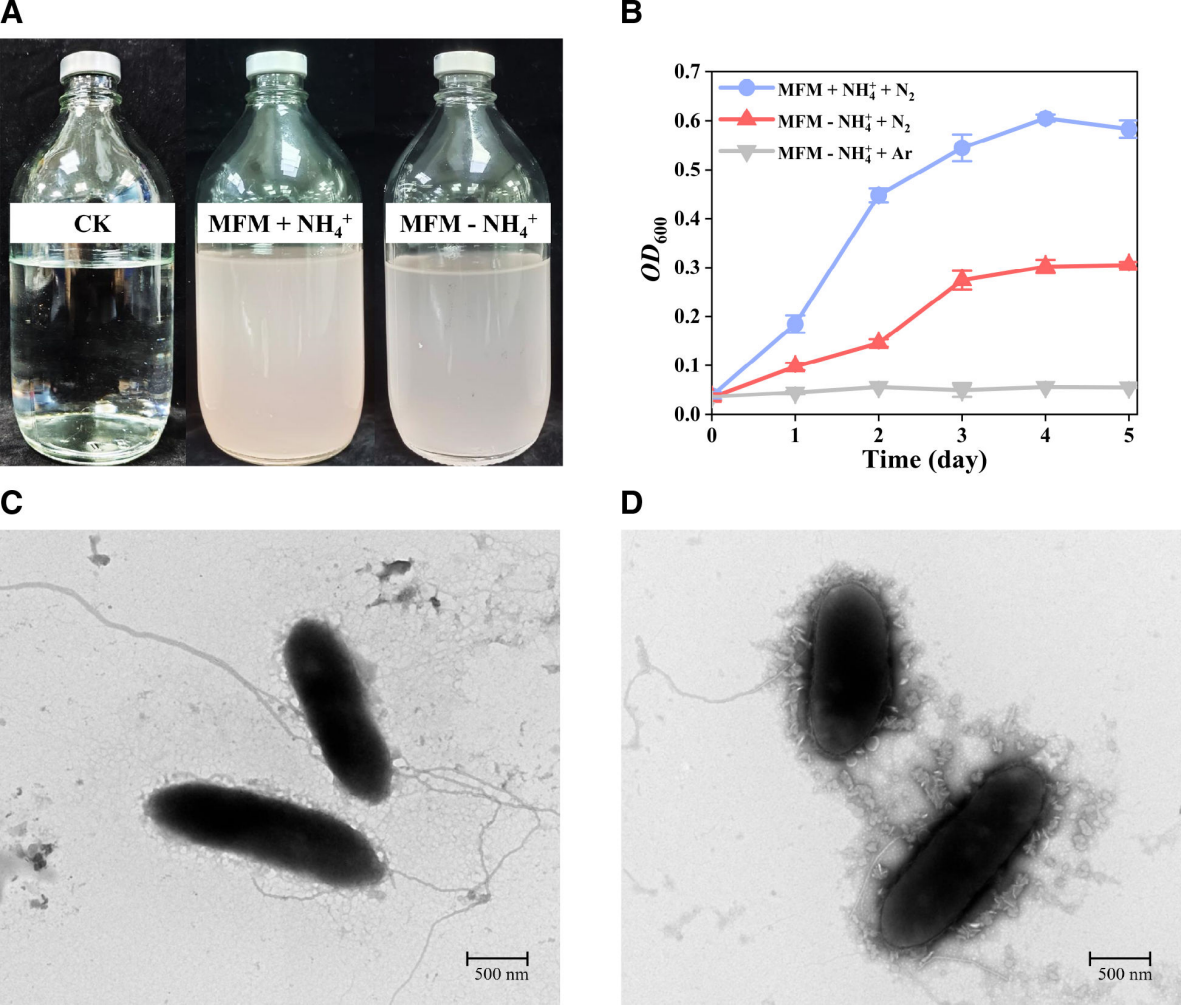
Biomass and cell morphology of strain *Geomonas nitrogeniifigens* RF4 cultured in MFM with and without NH_4_^+^. (**A**) Visual growth of strain *G. nitrogeniifigens* RF4; (**B**) growth curves of strain *G. nitrogeniifigens* RF4 grown under three conditions; (**C**) cell morphology of strain *G. nitrogeniifigens* RF4 cultured in MFM without NH_4_^+^; (**D**) cell morphology of strain *G. nitrogeniifigens* RF4 cultured in MFM with NH_4_^+^.

*G. nitrogeniifigens* RF4 grew best with 20 mM acetate and 10 mM glucose simultaneously as electron donors in comparison to acetate as the sole electron donor (Fig. S5). Hence, we chose two electron donors to perform assays in the following studies. In the absence of NH_4_^+^ addition, *G. nitrogeniifigens* RF4 grew well using N_2_ as the sole nitrogen source, indicating RF4 was able to fix nitrogen. Moreover, no growth was observed with Ar in replacement of N_2_. However, through the visual observations presented in Fig. 2A, *G. nitrogeniifigens* RF4 exhibited better growth cultured with NH_4_^+^ than without NH_4_^+^. The growth OD_600_ of *G. nitrogeniifigens* RF4 was more than twofold higher with NH_4_^+^ than with N_2_ after 4 days (Fig. 2B).

Morphological changes in cells can accompany different stresses such as high temperature (31) and oxidative stress (32). Compared to the non-nitrogen-fixing condition (with 1 mM NH_4_^+^), we observed significant differences in *G. nitrogeniifigens* RF4 viability, optical density, growth rate, and cell morphology under nitrogen-fixing conditions (no NH_4_^+^ added). For example, cells changed from being short rod-shaped (with NH_4_^+^) to long rod-shaped under nitrogen-fixing conditions (Fig. 2CD). Flagella under nitrogen-fixing conditions became longer and thicker, increasing their ability to obtain energy for survival.

**Fig 3:**
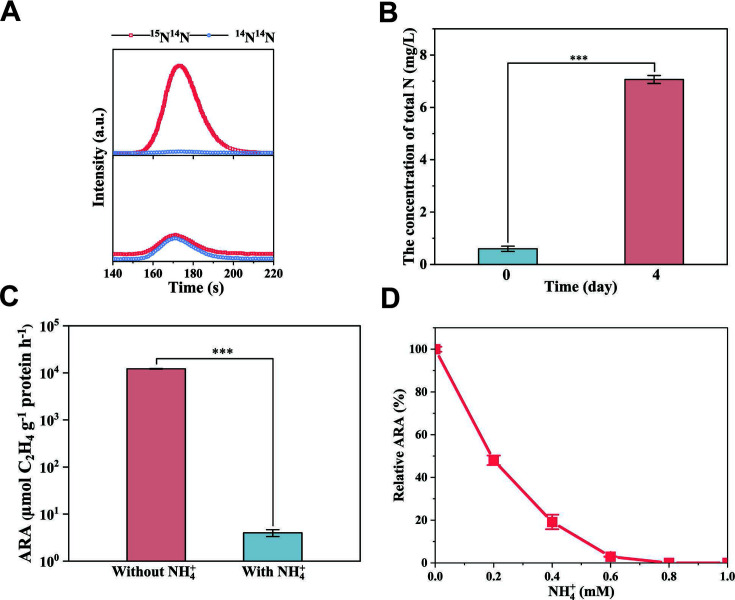
Nitrogen fixing ability and inhibiting effect of NH_4_^+^ on nitrogenase activity of strain *Geomonas nitrogeniifigens* RF4. (**A**) Elemental analysis-iso topic ratio mass spectrometry of ^15^N^14^N and ^14^N^14^N; (**B**) total nitrogen accumulation through nitrogen fixation; (**C**) nitrogenase activity estimated by the ARA method; (**D**) influence of NH_4_^+^ on nitrogenase activity.

To further confirm nitrogen fixation ability, ^15^N_2_ isotope labeling was used. Isotope mass spectrometer detection showed that the ratio of ^15^N/^14^N approximated 50.0135% under the nitrogen-fixing conditions, but ^15^N/^14^N was only 0.366% in the presence of 1 mM NH_4_^+^ (Fig. 3A). It was, indeed, confirmed that strain *G. nitrogeniifigens* RF4 was able to transform ^15^N_2_ to nutrients, thereby providing the necessary energy for its growth. To evaluate the nitrogen-fixing ability of *G. nitrogeniifigens* RF4, the total nitrogen concentration (TNC) was measured. When using N_2_ as the sole nitrogen source, we observed a significant 11.7-fold increase in TNC (increased to 6.4 mg L^−1^) after culturing for 4 days (Fig. 3B), indicating strain *G. nitrogeniifigens* RF4 was, indeed, able to fix N_2_ and convert it into nutrient nitrogen.

The nitrogen-fixing activity was estimated by ARA. In the absence of NH_4_^+^, ARA of *G. nitrogeniifigens* RF4 reached ~1.22 × 10^4^ µmol C_2_H_4_ g^−1^ protein h^−1^ (Fig. 3C). ARA was 4 µmol C_2_H_4_ g^−1^ protein h^−1^ in the presence of 1 mM NH_4_^+^. The nitrogen-fixing activity of strain *G. nitrogeniifigens* RF4 decreased as the NH_4_^+^concentration in the medium increased and was completely inhibited by 0.8 mM NH_4_^+^ (Fig. 3D). This was consistent with previous reports showing that NH_4_^+^ inhibited nitrogenase activity of diazotrophs such as *Geobacter* (33) and *Anaeromyxobacter* (2). However, NH_4_^+^ was not detected in the culture of *G. nitrogeniifigens* RF4 under nitrogen fixing conditions, indicating ammonia produced by nitrogen fixation process was almost completely used for the growth of *Geomonas*.

### Transcriptomic analysis of *Geomonas* under nitrogen-fixing conditions

Transcriptomic analysis was performed to determine the gene expression differences between nitrogen-fixing and non-nitrogen-fixing conditions. The clean data obtained from the cDNA libraries constructed from each sample under nitrogen-fixing and non-nitrogen-fixing conditions reached at least 3.08 Gb, with Q30 > 95.25%. More than 97% of the total reads were mapped to genes of the reference strain (Table 1). Principal component analysis (PCA) showed that the same treatment clustered together, indicating that the sequence data were reasonable and could be used for further studies (Fig. S6).

**TABLE 1:** Overview of RNA-seq statistics^*a*^

Information	Nitrogen fixation			Non-nitrogen fixation		
	1	2	3	1	2	3
Raw reads	31,998,330	26,958,666	27,174,476	26,210,342	29,159,980	29,339,978
Raw bases (bp)	4,831,747,830	4,070,758,566	4,103,345,876	3,957,761,642	4,403,156,980	4,430,336,678
Raw error rate (%)	0.0266	0.027	0.0278	0.028	0.029	0.0297
Raw Q20 (%)	96.9	96.67	96.26	96.22	95.66	95.28
Raw Q30 (%)	93.23	92.87	92.31	92.21	91.57	91.15
Clean reads	30,941,992	25,907,498	26,127,568	24,668,088	26,505,924	26,001,044
Clean bases (bp)	3,725,847,344	3,076,774,388	3,243,000,299	3,135,675,274	3,262,575,955	3,193,629,131
Clean error rate (%)	0.0232	0.0234	0.0239	0.024	0.0237	0.0237
Clean Q20 (%)	98.67	98.59	98.39	98.31	98.44	98.46
Clean Q30 (%)	96.05	95.89	95.41	95.25	95.56	95.63
Genome mapped ratio (%)	99.14	99.18	97.73	97.25	97.73	97.19

^*a*^ Note: 1, 2, and 3 represent three replicates for each experiment.

**Fig 4:**
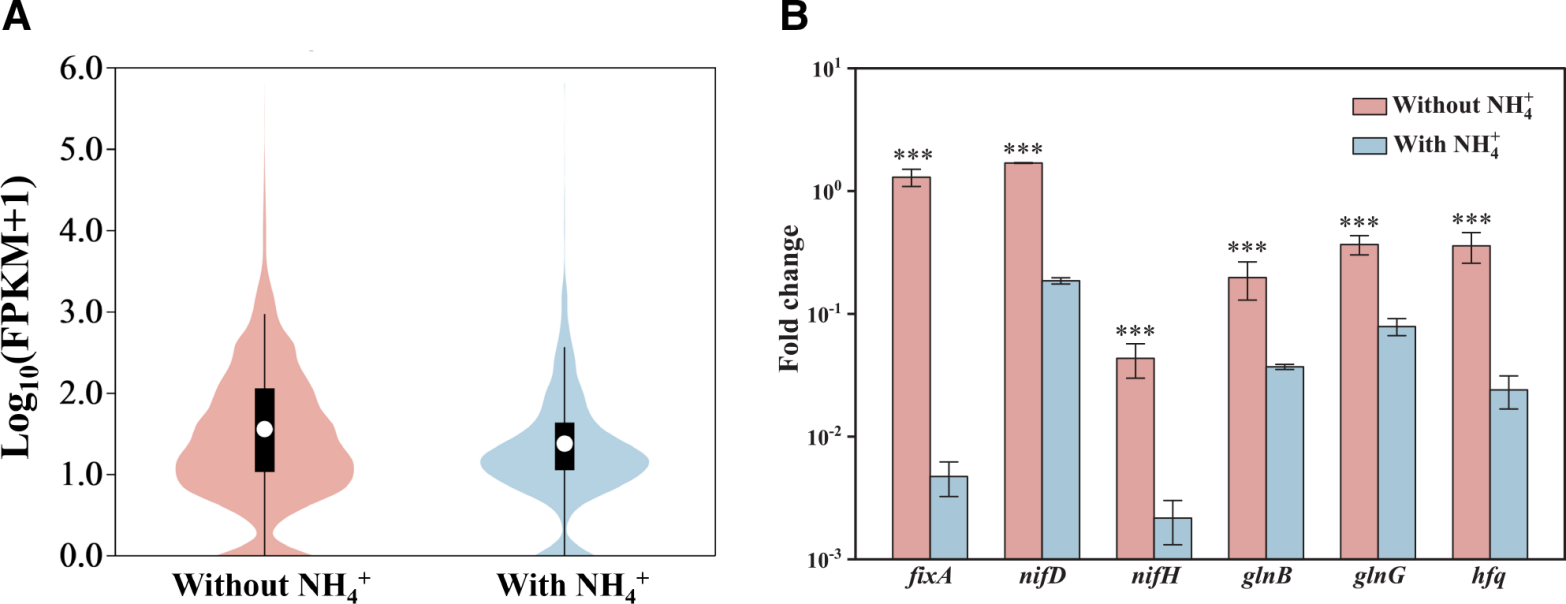
Differential expression levels (**A**) and selected differentially expressed genes confirmed by RT-qPCR (**B**) of strain *Geomonas nitrogeniifigens* RF4 when comparing growth with and without NH_4_^+^.

Using soft DESeq2, we defined differential expression as a log_2_-fold change of |log_2_FC| ≥ 1.00 and *P* < 0.05. A total of 1,826 genes were significantly differentially expressed, including 950 downregulated genes and 876 upregulated genes under nitrogen-fixing conditions. Gene expression under nitrogen-fixing and non-nitrogen-fixing conditions exhibited significant differences (Fig. 4A). We grouped these differently expressed genes by metabolic function in the KEGG database: 1,247 annotated genes were obtained with 956 genes enriched into 167 pathways (Fig. S7). Among the six major classifications of KEGG, the number of genes involved in metabolism was the highest. Under the metabolism classification, carbohydrate, energy, cofactors, and vitamins and amino acid metabolism contained more genes. Under cellular process classification, cellular community and cell motility contained more genes, flagella assembly encoding genes were most up regulated under nitrogen-fixing conditions. These genes might play an important role in the nitrogen fixation process of strain *G. nitrogeniifigens* RF4.

As expected, expression of most genes associated with nitrogen fixation increased under nitrogen-fixing conditions. Nitrogenase enzyme encoding genes *nifHDK* (log_2_FC = 2.42, 1.60, 1.35, respectively) were upregulated, while *nifEX* critical to N_2_ fixation exhibited upregulation but not significantly (*P* > 0.05). Nitrogen fixation-related genes *glnG* and *hfq* were upregulated (log_2_FC = 1.44, 2.37, respectively), which helped regulate and express genes for N_2_ fixation at low-nitrogen level. The ammonia transporter encoded gene *amtB* was more highly expressed (log_2_FC = 1.39), *glnAB* and *gltS* (log_2_FC = 1.38, 1.98, 0.39) associated with NH_4_^+^ uptake and transformation were upregulated under nitrogen fixation. The transcript level of glutamate dehydrogenase (*gdhA*) was downregulated in nitrogen-fixing cells but did not display a significant difference (*P* > 0.05).

The expression of genes encoding functions involved in electron transfer in *Geomonas* was also examined under nitrogen-fixing conditions. The electron bifurcating enzyme complex encoded by *fixAB* (log_2_FC = 4.41, 0.73, respectively) was upregulated. Ferredoxin (encoded by *fer*) was upregulated (log_2_FC = 4.02). Moreover, *por* encoding pyruvate: ferredoxin (flavodoxin) oxidoreductase was significantly upregulated, approximately fivefold. Gene expression of *nuoAB* encoding NADH-quinone oxidoreductase (log_2_FC = 2.02, 1.51, respectively) was high and upregulated. Additionally, flagellin (*fliC*, log_2_FC = 7.54) and flagellar motor protein (*motA*, log_2_FC = 3.03) were upregulated as well as *pilA* (log_2_FC = 4.15).

To verify the transcriptome analysis, the genes *glnB*, *glnG*, *hfq*, *fixA*, and *nifHD* were selected for RT-qPCR analysis. These genes encoded functions needed for nitrogen fixation such as nitrogenase, nitrogen regulators, and an electron transfer flavoprotein. Through the melting curve analysis, the products amplified were all single products, indicating that the primers used are of high quality. As shown in Fig. 4B, the results of RT-qPCR were consistent with the transcriptome analysis, which proved the reliability of the RNA-seq data.

**Fig 5:**
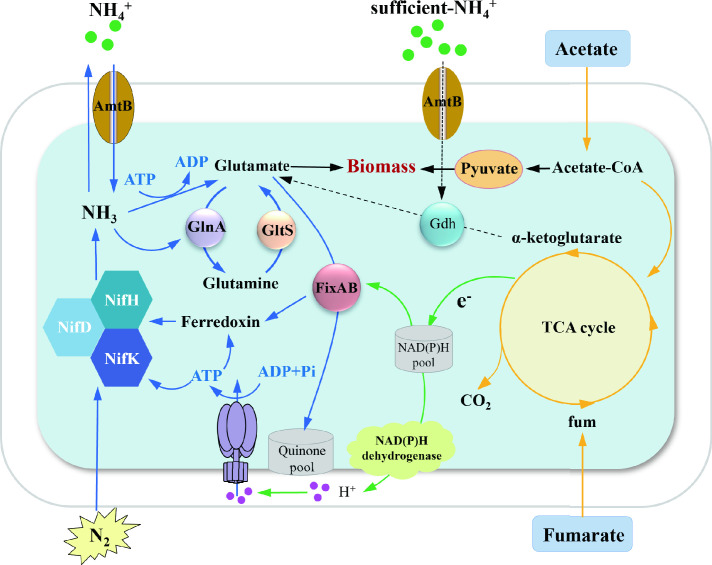
Proposed distribution of electron transfer and metabolic flow in *Geomonas* under nitrogen-fixing condition. Blue arrow, biological nitrogen fixation-related/upregulated pathway; green arrow, respiration/upregulated pathway; orange arrow, tricarboxylic acid cycle; black arrow, anabolism; dashed line, NH_4_^+^ sufficiency/downregulated pathways.

Based on the above analysis, we proposed a hypothetical scenario of nitrogen fixation pathways and their regulation in *Geomonas* under nitrogen-fixing conditions (Fig. 5). Nitrogen fixation and NH_4_^+^ assimilation by glutamine synthetase (GlnA) and glutamate synthase (GltS) were activated. Nitrogen-fixing genes such as *nifHD* were highly expressed. Cells need to obtain sufficient amounts of energy to generate electron donors such as ferredoxins/flavodoxins and ATP for nitrogen fixation.

## DISCUSSION

*Geomonas* has mainly been isolated from paddy soil. Despite it having been reported that *Geomonas* possesses the *nif* gene cluster (16, 17, 19), whether it is able to fix nitrogen in pure culture has remained unclear. In this study, we provide evidence to support the hypothesis that *Geomonas* is capable of N_2_ fixation.

Our data demonstrated that *Geomonas* species were abundant inhabitants of paddy soil. *nifH* was detected in all isolates, and the *nif* gene cluster was found on all sequenced genomes. The *nif* gene cluster structure in *Geomonas* was highly conserved, consisting of *nifHDKENBX*, *draG*, *fdxN,* and *draT* (Fig. 1B). Despite its close relation to *Geobacter*, *Geomonas* was shown to possess a distinct *nif* gene cluster. Compared to facultative anaerobic or strict anaerobic diazotrophs like *Anaeromyxobacter*, *Azotobacter*, *Pseudomonas*, *Pelobacter* (33–35), *Geomonas* exhibited a remarkably compact structure of a *nif* cluster and also contained the minimum required gene set for nitrogenase, *nifHDKENBX*. Furthermore, the essential residues of the active site of nitrogenase were also conserved (Fig. 1C). Phylogeny of the nitrogenase *nifHDK* nucleotide sequences revealed clear differences between *Geomonas* and *Geobacter* as well as *Anaeromyxobacter* (2). Thus, it is reasonable to suggest that the proteins encoded by the *nif* gene cluster in *Geomonas* form an active nitrogenase complex. However, the regulatory mechanism of nitrogen fixation in *Geomonas* requires further studies.

Exocellular electron transfer is a crucial process and the availability of an electron donor limits the biomass and growth rate of strict anaerobes (36, 37). Optimal electron donors were able to effectively increase the reducing reaction rate driven by anaerobes. Melo et al. (38) discovered that combing H_2_ and pyruvate resulted in faster reduction of 2,4-dinitroanisole (DNAN) compared to using only one electron donor. Similarly, Santos et al. (39) found that lactate was the most effective electron donor for sulfate removal in wastewater treatment with high sulfate levels. Previous reports indicated that acetate was preferred as the electron donor for *Geomonas* growth (17), but the resulting biomass was low (Fig. S5). The addition of carbon-rich substrates has been shown to stimulate growth and activity, increasing the demand for nitrogen (40, 41), glucose supplementation was able to promote a reduced rate of nitrogen fixation in the environment (42, 43). Our results indicated that combining acetate and glucose as electron donors significantly increased *Geomonas* growth.

The cell morphology has been shown to undergo changes to adapt to environmental stress. For example, *Rhodococcus* changed the cell shape in response to temperature variations (31), cyanobacterium displayed elongated cells under limiting phosphorus but sufficient nitrogen levels (44), and the flagella of *Ralstonia eutropha* changed according to nutrient supply (45). However, no reports have shown the morphological comparison of cells of anaerobes grown under nitrogen-fixing versus non-nitrogen-fixing conditions. In this study, we observed the cell morphology of *Geomonas* was changing including cell shape and flagella when cultured under nitrogen-fixing condition.

Although it has been reported that some *Geomonas* species contained the *nif* cluster and were able to grow under nitrogen-fixing conditions without ammonia (17), there have been no studies characterizing nitrogenase activity of *Geomonas in vitro*. Our studies clearly demonstrated that *Geomonas* was able to fix and assimilate N_2_ using nitrogenase. In addition, the nitrogenase activity of *Geomonas* was also suppressed by NH_4_^+^, a response that had been observed in other diazotrophs, including *Anaeromyxobacter* (2, 13, 46). Previous studies had displayed physiological features of *Geomonas*, such as iron reduction (16, 17), which was shown to play a crucial role in the rice soil ecosystem. The addition of ferric compounds has been predicted to enhance the nitrogen-fixing activity in paddy soil (47, 48). Our study revealed that nitrogen fixation was an important feature of *Geomonas*, suggesting its importance in rice soil ecosystems.

To understand the nitrogen fixation process in *Geomonas,* we examined gene expression by transcriptome analysis under nitrogen-fixing conditions. The *amt* gene, encoding an ammonium transporter, was activated at low nitrogen levels. When NH_4_^+^ concentrations are sufficient (>1 mM), ammonium can diffuse through the membrane as NH_3_ (49). Thus, we found *amt* expression was upregulated under nitrogen-fixing conditions, consistent with findings in *Geobacter* (29). The *hfq* gene was shown to regulate nitrogen fixation in other diazotroph (50) and was upregulated in *Geomonas* under nitrogen-fixing conditions. Glutamine synthetase encoded by *glnA* and glutamate synthase encoded by *gltS* are needed to synthesize the respective amino acids during anabolism providing a source for growth (51–53). We observed an upregulation of these genes in this study. *glnB* encoding the P-II family nitrogen regulator was highly expressed under nitrogen-fixing conditions (29). *gdhA* encoding glutamate dehydrogenase was predicted to be repressed during nitrogen fixation (54) similar to previous findings in *Geobacter* (27). However, *gdhA* was not significantly downregulated in our study.

Flavin-based electron bifurcation is an important energy coupling mechanism in the anaerobic microbial metabolism (55, 56), which involves the splitting of a hydride electron pair by flavoproteins into two separate electrons with different reduction potentials (56, 57). Genes encoding the flavin-based electron bifurcating enzyme complexes NfnAB and EtfAB have been observed in *Geobacter* (13, 29). In this study, we observed high expression of *fixAB* encoding flavin-based electron bifurcation complexes similar to EtfAB in *Geobacter*, suggesting FixAB system was involved in nitrogen fixation in *Geomonas*. The Fix system has also been identified in the diazotrophs *A. vinelandii* and *Rhodopseudomonas palustris*, which facilitates the reduction of ferredoxins or flavodoxins for N_2_ fixation (58–60). Jing et al. (13) reported that EtfAB and NfnAB in *Geobacter sulfurreducens* were upregulated in microbial electrolysis cells under nitrogen-fixing conditions, showing these complexes might reduce ferredoxins to drive N_2_ fixation. Ortiz-Medina et al. (29) found that *Geobacter* exhibited higher nitrogen fixation activity when gene expression of EtfAB was upregulated when exposed to a potential of −0.15 V. However, no previous study has investigated electron bifurcation in *Geomonas*. Our results suggest that nitrogen limitation promotes electron bifurcation providing substantial energy for N_2_ fixation.

Although strategies for generating reduced ferredoxins/flavodoxins for nitrogen fixation in *Geomonas* have not been studied, understanding these mechanisms is crucial. Ferredoxins (encoded by *fer*) are involved in respiration, nitrogen fixation, and carbon dioxide fixation in strict anaerobes (55). During nitrogen fixation, ferredoxin has been shown to act as an electron donor for nitrogenase (60, 61). We observed high expression of the *fer* gene in *Geomonas* under nitrogen-fixing conditions. In *Rhodospirillum rubrum*, pyruvate-ferredoxin oxidoreductases (PFOR) have been found to contribute modestly to nitrogen fixation (62). Although many diazotrophs possessed PFORs on their genomes, only a few were shown to support nitrogenase activity. We found that PFOR in *Geomonas* was upregulated under nitrogen-fixing conditions. However, further research is needed to confirm the contribution of PFOR to nitrogen fixation in *Geomonas*. Additionally, *fliC-*encoded flagellin, the major constituent of bacterial flagella, was significantly upregulated under nitrogen-fixing conditions. In contrast, flagella-related genes were downregulated in *Geobacter* during nitrogen fixation. Similar to *Geobacter*, *pilA* was not listed under the “electron transfer activity” category (29, 63) but might indirectly contribute to nitrogen fixation.

In conclusion, we demonstrated that *Geomonas* inhabited paddy soils and were the first to characterize the nitrogen-fixing properties of *Geomonas* at the cultural level. This research provides valuable insights into growth conditions, nitrogen fixation properties, and gene expression patterns associated with nitrogen fixation in *Geomonas*. These findings highlight the significance of *Geomonas* as a diazotrophic organism and its crucial role in nitrogen fixation in rice fields, thereby providing a new strategy for future reduction in the use of nitrogen fertilizer. Overall, our study contributes to a comprehensive understanding of *Geomonas* and its potential applications as a new pathway for reducing the dependency on nitrogen fertilizers in sustainable agriculture.

## MATERIALS AND METHODS

### Soil samples collection, *Geomonas* isolation, and identification

Paddy soil samples were collected from a rice field in Fujian Province, China (26.1076^°^ N, 119.3014^°^ E). No fertilizer had been applied to these paddy soils for at least 3 years. This soil was also used in the previous research (16). *Geomonas* strains were isolated and cultured as described before by Liu et al. (16). The single colonies obtained were purified and identified by their 16S rRNA gene sequence. Pairwise 16S rRNA gene sequence similarities between the isolates and the comparison to related type strains were calculated using the EzBioCloud platform (64). For phylogenetic analysis, 16S rRNA gene sequences of closely related type strains were downloaded from the EzBioCloud platform. Phylogenetic trees were constructed based on the neighbor-joining (65) method with the Kimura two-parameter model (66) and 1000 bootstrap replications (67) using MEGA version X (68).

### DNA extraction and genomic sequencing

For genome sequencing, genomic DNA was extracted from cells of *Geomonas* isolates cultured in R2A with 40 mM disodium fumarate using TIANamp Bacterial DNA kit (TIANGEN, China) according to the manufacturer’s instructions. Genomes were sequenced using Nanopore PromethION platform and Illumina NovaSeq PE150 at the Novogene Bioinformatics Technology Company, Ltd. (Beijing, China). The Kyoto Encyclopedia of Genes and Genomes (KEGG) database was used to predict gene functions (69, 70).

The nucleotide sequences of *nifHDK* of *Geomonas* and related strains were retrieved from the genomes sequenced in this study and the NCBI database. Phylogeny was inferred based on the neighbor-joining (65) method with 1000 bootstrap tests (67) using MEGA version X (68). Structural schematic diagram of the nitrogenase was drawn online using ChiPlot (https://www.chiplot.online/gene_cluster.html) to compare the differences in nitrogenase structure between *Geomonas* and related diazotrophic species.

### Growth and cell morphology

*Geomonas nitrogeniifigens* RF4 was cultured in MFM broth. To optimize the growth conditions for *G. nitrogeniifigens* RF4, electron donors at different concentrations were tested in the modified MFM broth. The modified MFM (L^−1^) was composed of 2.0 g NaHCO_3_, 0.2 g MgSO_4_·7H_2_O, 0.3 g KH_2_PO_4_, 0.16 g CaCl_2_·2H_2_O, 1.0 mL vitamin stock solution (L^−1^, 0.02 g biotin, 0.05 g folic acid, 0.1 g pyridoxine-HCl, 0.05 g thiamine-HCl, 0.05 g nicotinic acid, 0.05 g aminobenzoic acid, 0.05 g Ca-pantothenate, 0.01 mg vitamin B12, and 0.05 g lipoic acid), and 1.0 mL mineral stock solution [L^−1^, 1.5 g nitrilotriacetic acid, 3.0 g MgSO_4_, 0.1 g FeSO_4_·7H_2_O, 0.5 g MnSO_4_·H_2_O, 1.0 g NaCl, 0.1 g CoCl_2_·6H_2_O, 0.1 g CaCl_2_·2H_2_O, 0.13 g ZnCl_2_, 0.01 g CuSO_4_·5H_2_O, 0.01 g AlK(SO_4_)_2_·12H_2_O, 0.01 g H_3_BO_3_, 0.025 g Na_2_MoO_4_·2H_2_O, 0.024 g NiCl_2_·6H_2_O, 0.025 g Na_2_WO_4_·2H_2_O]], and supplemented with 20 mM sodium acetate and 40 mM disodium fumarate. The pH was adjusted to 6.5. To observe the changes in cell morphology of *Geomonas* under nitrogen-fixing and non-nitrogen-fixing conditions, strain RF4 was cultured in modified MFM with 1 mM NH_4_Cl or without NH_4_Cl but with N_2_ as the sole nitrogen source at 30°C for 3 days without shaking. The cultured cells were observed by transmission electron microscope (Hitachi HT7700, Japan) using the negative staining method with 1% phosphotungstic acid.

### Assays of N_2_ fixation and nitrogenase activity

Under nitrogen-fixing conditions, the optimal cell growth conditions of strain RF4 were determined by growth curves (OD_600_). Two milliliters of strain RF4 culture in log phase in modified R2A (R2A + 20 mM disodium fumarate) was centrifuged for 10 min at 4,500 × *g*. To estimate nitrogen-fixing ability and nitrogenase activity, the cells were washed three times with oxygen- and nitrogen-free sterile MFM broth and then transferred into MFM with and without ammonia and incubated for 3 days without shaking, respectively. The electron acceptor under both treatments was fumarate, and acetate and glucose were both provided as electron donors. As a control experiment, the same system was set up using pure Ar instead of N_2_/CO_2_ gas. The strain was anaerobically incubated in serum bottles sealed with butyl rubber plug and aluminum crimp under N_2_/CO_2_ (80:20 [vol/vol]) atmosphere at 30 ℃ without shaking. Three replicates were set for each experiment in this study.

Nitrogen fixation activity was measured by the ARA based on acetylene (C_2_H_2_) reduction into ethylene (C_2_H_4_) by nitrogenase (71). Briefly, strain RF4 was precultured in the modified MFM medium obtained from the analysis described above for 4 days. The experimental conditions were identical except the head space in the bottle was Ar/C_2_H_2_ (90:10 [vol/vol]) gas. The negative control was set up using pure Ar gas. C_2_H_4_ production was measured by gas chromatography on a fused-silica column (Porapak; Hychrom) (16). The inhibitory effect of NH_4_^+^ on nitrogen fixation activity was determined in N-free MM with 0–1 mM NH_4_Cl added (interval of 0.2). The effect of different concentrations of ammonium on nitrogen fixation activity was estimated based on ARA as described above. The nitrogen-fixing rate was estimated by calculating the amount of total nitrogen during the nitrogen fixation process. The total nitrogen was extracted using total nitrogen detection kit (HACH) according to manufacturer’s instruction and measured on a UV Spectrophotometer (DR3900, HACH).

To further confirm whether strain RF4 facilitated nitrogen fixation, an isotope labeling enrichment assay was conducted as described previously (13). Briefly, 2 mL cell culture of strain RF4 in log phase cultured in modified R2A was centrifuged for 10 min at 4,500 × *g*. The cells were washed three times with oxygen-free sterile MFM broth (without N) and then transferred into ^15^N_2_ serum bottles and incubated for 3 days without shaking. The serum bottle contained 20 mL sterile N-free MFM (without NH_4_^+^) and MFM (with 1 mM NH_4_^+^), respectively. The headspace of a serum bottle containing N-free MFM was first full of Ar, and then 10% vol was replaced by ^15^N_2_. The cells were harvested and freeze-dried for further ^15^N isotope analysis. The δ^15^N value (^15^N [*m/z* = 29]/^14^N [*m/z* = 28]) of bacterial cells was determined by an isotope ratio mass spectrometer (EA-IRMS, Thermo Scientific MAT253 Plus gas bench isotope mass spectrometer).

### RNA extraction and transcriptome sequencing

Strain RF4 was cultured in MFM medium with NH_4_^+^ or without NH_4_^+^ for 3 days. The cultures were collected by centrifugation at 10,000 × *g* for 2 min. The cells were frozen in liquid nitrogen and stored at −80°C for subsequent RNA extraction. RNA was extracted using TRIzol Reagent kit according to the manufacturer’s instructions (Invitrogen). RNA concentration and purification were detected by NanoDrop 2000 (Thermo Scientific). Transcriptome sequencing was performed by Majorbio Bio-pharm Technology Co., Ltd (Shanghai, China). All statistical analysis was performed using the free online platform of Majorbio Cloud Platform (https://www.majorbio.com).

### RT-qPCR

Fluorescence real-time quantitative PCR (RT-qPCR) was performed to evaluate the reliability of RNA-Seq transcriptome results. By comparing the expression of genes encoding functions involved in the nitrogen fixation process, a total of six upregulated genes were selected for RT-qPCR analysis (Table S2). RNA was isolated using the same process as in the transcriptome analysis. Double-stranded cDNA was synthesized using a SuperScript double-stranded cDNA synthesis kit (Invitrogen, CA). RT-qPCR was performed using 2× Taq Plus Master Mix by following the manufacturer’s protocol. For genes *glnB*, *glnG*, *hfq, fixA*, and *nifHD*, *rpoB* was used as internal reference for quantitative analysis of relative expression. The thermal cycling for all genes was performed as follows: preheat at 95°C for 300 s followed by 35°C cycles of 95°C for 30 s, 52°C for 30 s, and 72°C for 60 s. Three biological replicates were used in this study.

## Data Availability

The transcriptomic data described in this article has been deposited in the NCBI Sequence Read Archive under SRA accession number SRP460626.

## SUPPLEMENTAL MATERIAL

Supplemental material mbio.02150-23-s0001.pdf Tables S1 and S2 and Fig. S1 to S7.

ASM does not own the copyrights to Supplemental Material that may be linked to, or accessed through, an article. The authors have granted ASM a non-exclusive, world-wide license to publish the Supplemental Material files. Please contact the corresponding author directly for reuse.
