# Supplementary material for: Nitrogen fixation and transcriptome of a new diazotrophic Geomonas from paddy soils
Source: mBio. 2023 Oct 19;14(6):e02150-23. doi: 10.1128/mbio.02150-23 (PMC10746287; doi:10.1128/mbio.02150-23)
Supplement: Supplemental material — Tables S1 and S2 and Fig. S1 to S7. [file mbio.02150-23-s0001.pdf]

Nitrogen fixation and transcriptome of a new diazotrophic *Geomonas* from paddy soils

Guo-Hong Liu[1], Shang Yang[2], Shuang Han[2], Cheng-Jie Xie[2], Xing Liu[2], Christopher Rensing[2#] Shun-Gui Zhou[2#]

[1]Institute of Resources, Environment and Soil Fertilizer, Fujian Academy of Agricultural Sciences, Fuzhou City, Fujian Province, 350003, PR China.

[2]Fujian Provincial Key Laboratory of Soil Environmental Health and Regulation, College of Resources and Environment, Fujian Agriculture and Forestry University, Fuzhou City, Fujian Province, 350002, PR China.

***Authors for correspondence:**

Shun-Gui Zhou

Tel & Fax: +86 591 86397843

E-mail: sgzhou@fafu.edu.cn

Christopher Rensing

E-mail: crensing94@gmail.com

Table S1 Genome information for the genus *Geomonas*

Species	Contigs	Size (bp)	CDS nmber	rRNA	tRNA	DNA G+C (%)
Geomonas agri	7	4684271	4030	7	54	60.74
Geomonas anaerohicana	48	5078966	4290	3	57	61.63
Geomonas azotofigens	18	4973295	4225	7	53	62.43
Geomonas bemidjiense	1	4615150	4019	12	60	60.27
Geomonas bremense	1	4458493	3889	12	62	60.54
Geomonas diazotrophica	58	4651047	3980	6	62	61.94
Geomonas edaphica	17	4758995	4127	6	55	60.45
Geomonas ferrireducens	14	4808514	4171	5	58	60.67
Geomonas fuzhouensis	25	5130833	4455	5	54	61.62
Geomonas limicola	17	5233656	4416	4	53	61.82
Geomonas nitrogeniifigens RF4^{T}	1	4829642	4129	12	62	61.74
Geomonas oryzae	18	4933374	4206	7	57	61.24
Geomonas oryzisoli	1	4846465	4101	12	60	61.77
Geomonas paludis	30	5114951	4253	6	58	62.41
Geomonas propionica	58	5000662	4339	3	53	61.17
Geomonas silvestris	36	5118299	4310	7	60	62.61
Geomonas subterranea	1	4991523	4305	12	62	61.89
Geomonas terrae	7	4696900	4008	4	53	60.97
Geomonas sp. RF6	1	5258610	4405	12	64	61.30
Geomonas paludis RG22	1	5241294	4339	12	62	62.20
Geomonas nitrogeniifigens RG29	1	4761015	4043	12	61	61.95
Geomonas paludism RG31	1	5107768	4179	12	60	62.57
Geomonas subterranea RG3	1	4991984	4256	12	62	61.90

Table S2 Primers for RT-qPCR tests used in this study

Gene	Primer	Sequences（5′-3′）	Reference
nifH	*nifH* -F	TGCGAYCCSAARGCBGACTC	1
	nifH -R	ATSGCCATCATYTCRCCGGA	
nifD	*nifD* -F	CCACATCTCCAACGACACCA	In this study
	nifD -R	AGCAGTGGATCAGGTTCAGC	
rpoB	*rpoB* -F	CTGCGAGGAACCGAAGAACT	In this study
	rpoB -R	GAGCTGAAAAACGGCAAGGG	
glnB	F	CGGTCGTCAGAAGGGTCA	In this study
	R	CGCCGATGCGTCCAGTTT	
glnG	F	CGAGGAATACGAACTGCTGC	In this study
	R	CTCGATGGCGTTCTGGGT	
hfq	F	CAGGACCAGTACCTCAACC	In this study
	R	CAGTCGGAACACGCCATC	
fixA	F	TCTGCGGCAAACAGACCAT	In this study
	R	TGTCCCAGACCTTGACCTCC	
16S rRNA	F	ACTCCTACGGGAGGCAGCAG	2
	R	GGACTACHVGGGTWTCTAAT	

Reference:

1 Zhuang W, Yu X, Hu R *et al*. 2020. Diversity, function and assembly of mangrove root-associated microbial communities at a continuous fine-scale. npj Biofilms Microbiomes 6, 52. https://doi.org/10.1038/s41522-020-00164-6

2 Dai T, Wen D, Bates CT, Wu L, Guo X, Liu S, Su Y, Lei J, Zhou J, Yang Y. 2022. Nutrient supply controls the linkage between species abundance and ecological interactions in marine bacterial communities. Nat Commun 13, 175. https://doi.org/10.1038/s41467-021-27857-6

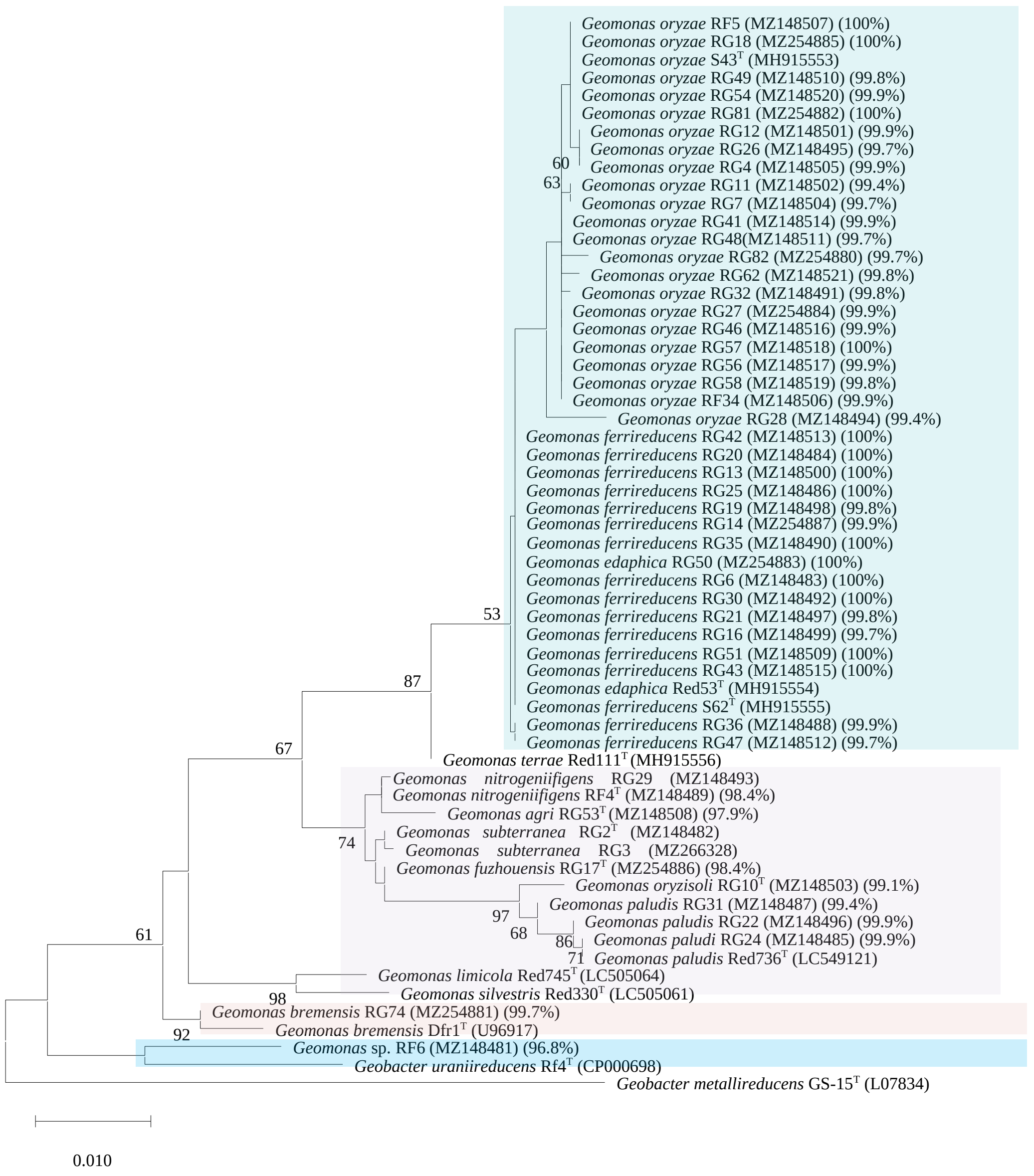

Fig S1 Phylogeny of diazotrophic genus *Geomonas* from paddy soil based on 16S rRNA gene. Branches corresponding to partitions reproduced in less than 50% bootstrap replicates are collapsed. The significance of each branch is indicated by a bootstrap value calculated for 1000 subsets. Bar, 0.01 substitutions per site. The content in the bracket was the accession number of 16S rRNA gene sequence of each strain. The information in each branch was in order of species name, strain code, 16S rRNA gene accession number, 16S rRNA gene similarity between isolate and its closest type strain.

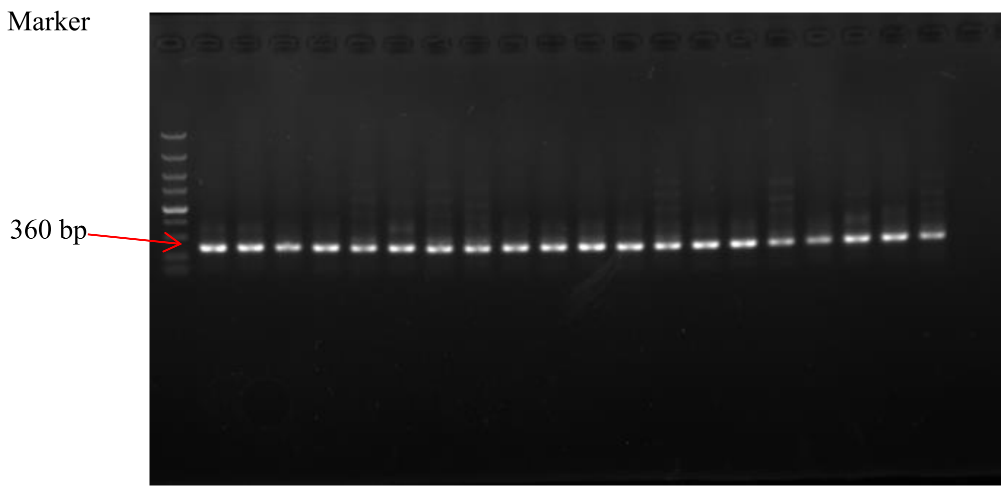

Fig S2 *nifH* gene amplification of the representative strains of the genus *Geomonas*

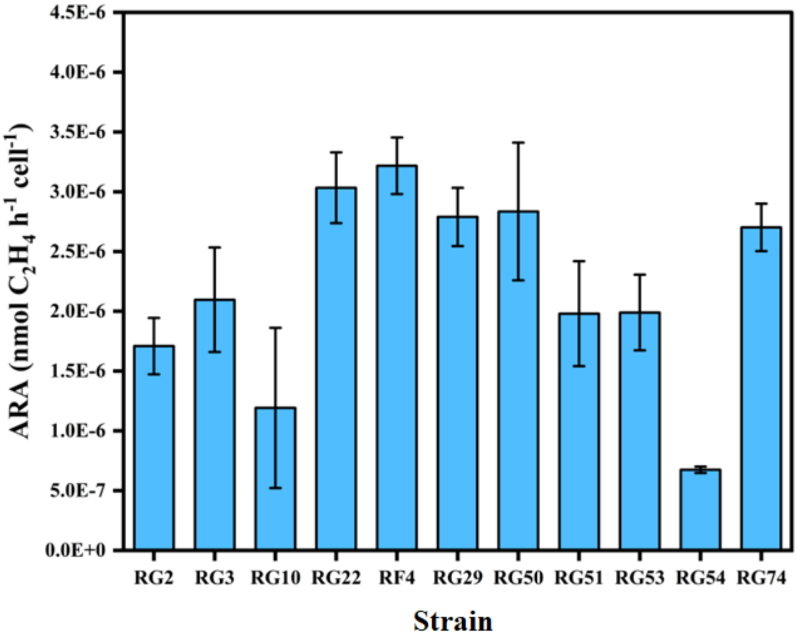

Fig S3 Nitrogenase activity of representative *Geomonas* strains grown for 24 h and measured by ARA.

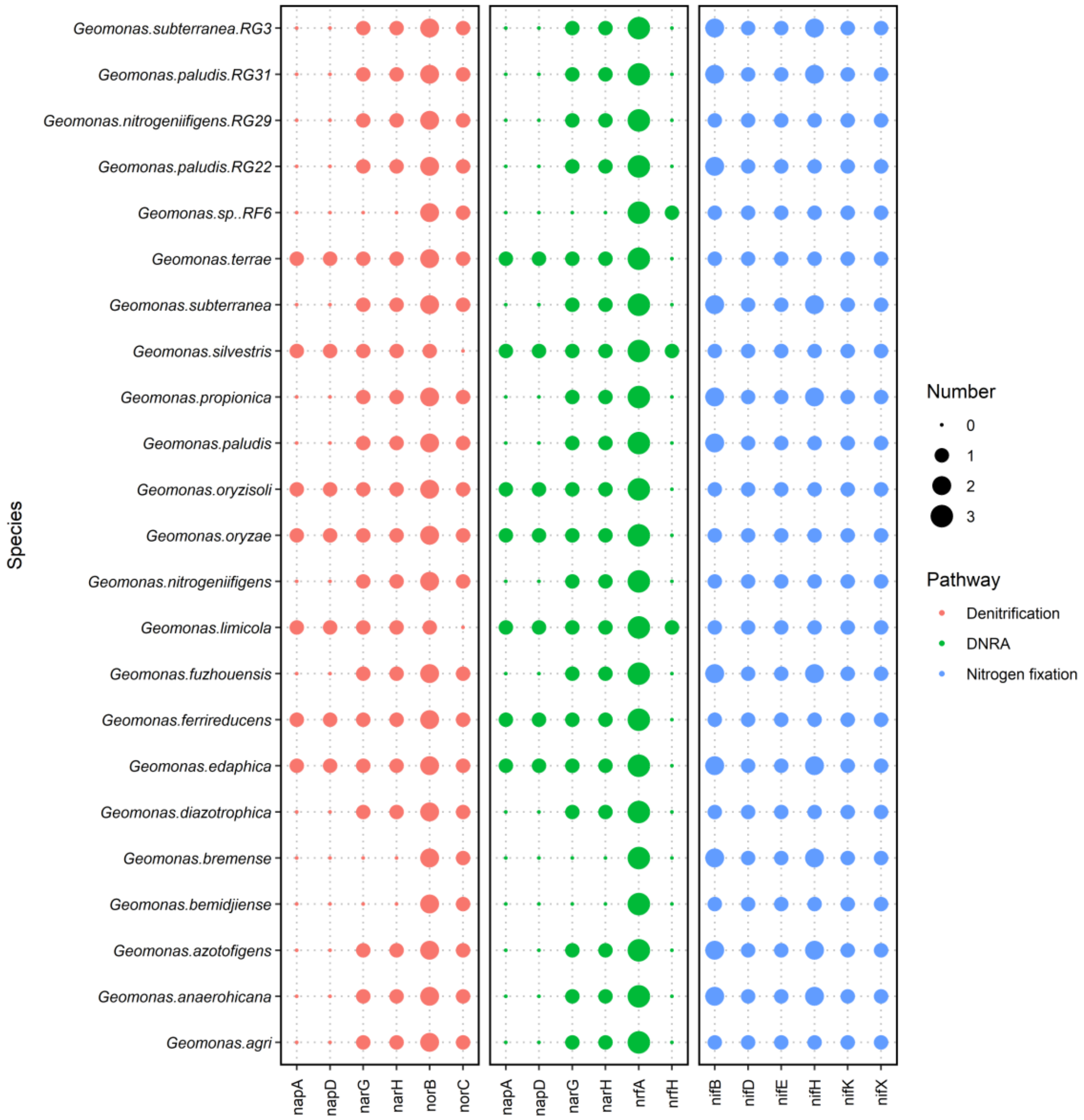

Fig S4 Nitrogen cycling related genes of *Geomonas* based on genome prediction with KEGG. DNRA stands for Dissimilatory Nitrate Reduction to Ammonia.

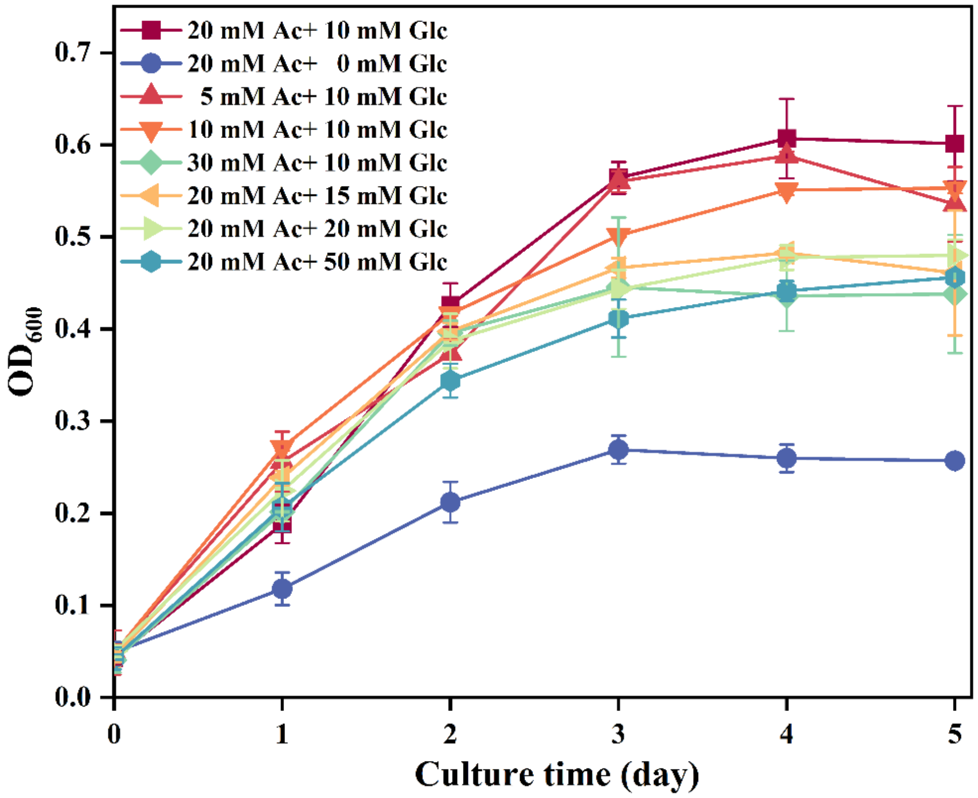

Fig S5 Growth condition optimization of strain *Geomonas nitrogeniifigens* RF4 using different concentrations of electron donors in 2×MFM broth. Glc: glucose; Ac: acetate. All data presented was the averaged values with standard deviations (SD) calculated from triplicate measurements.

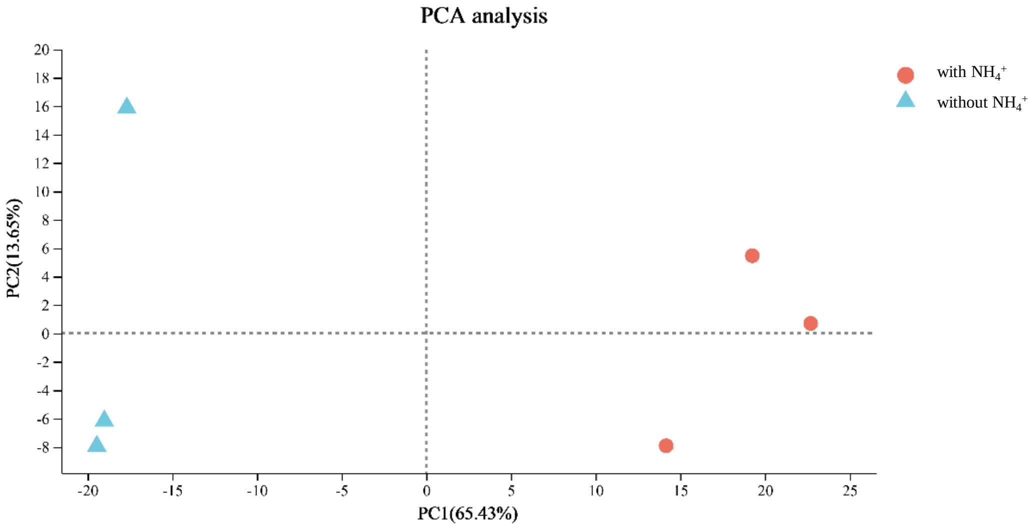

Fig S6 PCA (Principal Components Analysis) of gene expression of strain *G. nitrogeniifigens* RF4 under nitrogen-fixing and non-nitrogen-fixing conditions.

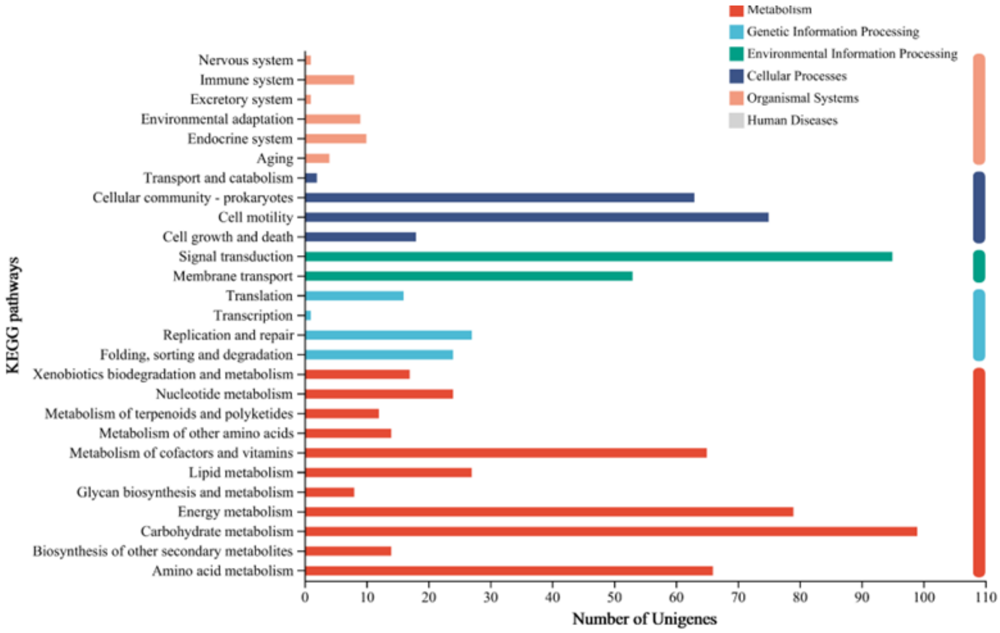

Fig S7 KEGG annotation of strain *G. nitrogeniifigens* RF4 under nitrogen-fixing and non-nitrogen-fixing conditions
